# Assessing dental students’ professional satisfaction with operative dentistry teaching and curriculum: A study in Saudi Arabia

**DOI:** 10.1097/MD.0000000000026459

**Published:** 2021-06-25

**Authors:** Mohammed A. Alqarni

**Affiliations:** Department of Restorative Dentistry, King Khalid University College of Dentistry, Director of Saudi Board of Restorative Dentistry Training Centers at Southern Province, P.O. Box 3263, Abha, Kingdom of Saudi Arabia.

**Keywords:** curriculum, dental students, operative dentistry, professional training satisfaction

## Abstract

Evaluating students’ professional training satisfaction with operative dentistry teaching and curriculum can help identify their educational needs and improve the quality of the education imparted. This study aimed to assess the professional training satisfaction of senior undergraduate dental students in Saudi Arabia from the operative dentistry course teaching and its curriculum at different levels and among genders.

A total of 193 (109 male, 56.48%; and 84 female, 43.52%) students participated in the survey. The respondents were at the 10th, 11th, and 12th levels of the Operative Dentistry course in a ratio of 34.2%, 32.1%, and 33.7%, respectively. Data were collected from survey items (18 questions) covering 6 areas: learning objectives, course materials, content relevance, instructor knowledge, instructor delivery and style, and facility and environment. Descriptive and analytical tests were performed using SPSS Software 19, with the significance level set at 0.05.

A high level of satisfaction was seen among level 10 (68.18%), 11 (79.03%), and 12 (86.15%) students. A significant statistical difference was observed among level 10 students with a low level of satisfaction and a high level of satisfaction (*P* = .045). The percentage of satisfaction increased with the level. A high level of satisfaction was seen among male (78.90%) and female (76.19%) students, with a total satisfaction level of 77.72%.

Continuous evaluation and assessment of teaching and curriculum can be a tool to improve the quality of education imparted, especially in clinical courses such as operative dentistry. This helps to prepare students for their professional life as healthcare providers. The role of teaching skills related to amalgam must be re-evaluated. It is recommended to include student representation and participation in course development committees, as they are the final recipients of the educational process.

## 1 Introduction

Any training program needs evaluation for quality assurance, and further improvement.^[1]^ Kirkpatrick's model of evaluation proposes 4 increasing levels to assess the impact of training programs. Level 1 (*reaction*) measures how the person feels about a course; level 2 (*learning*) measures the extent to which principles, facts, and techniques have been understood and absorbed; level 3 (*behavior*) measures the application of the principles and techniques acquired on the job; and level 4 (*results*) measures the ends, goals, and results desired.^[1,2]^ Monitoring students’ reaction to their learning experiences is increasingly being undertaken by higher education institutions.^[3]^ This initial level of evaluation should be an inherent feature of every training program because it indicates how a training program can be enhanced and further developed besides building the base for higher levels of evaluation since reactions serve as a pointer to whether learning is possible.^[1,2]^ Students’ satisfaction with and attitudes toward training programs are the most common indicators used to assess the reaction.^[1,3]^ However, there is additional value in exploring graduates’ reactions to training programs because they are less emotionally attached to the institution and are back at their workplaces where they can judge whether the knowledge and skills acquired during the program match their job requirements and responsibilities.

The undergraduate dental curriculum “should prepare graduates to enter practice.”^[4]^ The course's primary aim is to help students with their career expectations and develop their identity as a professional workforce. The secondary aim is to teach skills that students can use when applying for a job.^[5]^ Satisfaction and attitude are 2 indicators that help determine education quality. Satisfaction refers to the level to which students’ experiences meet their expectations.^[6]^ However, attitudes refer to a mixture of beliefs, thoughts, and feelings that predispose graduates to respond positively or negatively toward institutions.^[7,8]^ In addition to their role in ensuring learning and teaching quality standards, the 2 indicators serve as a guide for students to aid their decision-making at the program/institution level and compute institutional performance indicators.^[9]^

The format of dental education varies across the world, while some institutions have a yearly system, others follow a semester system. Several factors affect the learning process in any course, such as the nature of the student him/herself, the quality of demonstrators and teachers, exposure to course materials, laboratory, clinical facilities, and so on. Continuous evaluation of these factors is necessary to ascertain whether the conditions are conducive to learning in the assessed program.

Operative dentistry is an essential branch of dentistry, constituting a significant part of the teaching process in dental colleges. Students are introduced to operative dentistry from level 4, and this continues until level 12. There has been continuous development in the field of operative dentistry, both in terms of materials, equipment, and techniques. The students attend various continuing dental education programs conducted by national dental organizations and professional societies during their course, where they are exposed to newer materials, techniques, and various treatment protocols. In addition, the availability of this information is also available on social media and other online platforms. Thus, the present generation of students have a high level of awareness and expectations. Hence, in the present scenario, it is imperative to evaluate the students’ level of satisfaction and to correlate and implement the findings to develop the course. The operative dentistry course teaching in the dental school undergoes a review every semester, and improvements are suggested and implemented to keep students abreast of the latest. It is vital to analyze and understand whether the changes made to the course achieve the intended goals. As with all recent developments, continuous assessment of teaching strategies is necessary for a comprehensive evaluation of the teaching program.

One of the methods for evaluating an educational system is surveying student opinions because students experience the teaching's full effect during the course.^[10]^ As the primary recipients of the educational system, evaluating student satisfaction in professional training is one of the significant components of assessing and improving the quality and delivery of education.^[11,12]^ Many studies have been reported regarding dental students’ satisfaction, perception, attitude, and career motivations in general.^[13–17]^ The learning process is different for each individual; even in the same educational environment, learning may not occur in all students at a similar level and quality.^[18]^ This difference may be attributed to students’ different backgrounds, strengths, weaknesses, interests, ambitions, levels of motivation, and approaches to studying.^[19]^ Student satisfaction definitions have been interpreted widely in different ways. Elliott and Healy defined student satisfaction as a “short-term attitude resulting from an evaluation of a student's educational experience” and claimed that student satisfaction was achieved when their actual experiences met or exceeded their initial expectations.^[20]^ Aldridge and Rowley divided student satisfaction evaluations into 2 categories, focusing on classroom teaching and learning evaluation and the second being focused on the comprehensive student experience.^[21]^ Bryant and Bodfish claimed that student satisfaction was a significant performance indicator for higher education institutions, with many universities implementing rigorous quality assurance processes.^[22]^ Most colleges measure student satisfaction by administering student satisfaction surveys such as the Freshmen Survey, National Survey of Student Engagement, Student Strength Inventory, and Noel Levitz survey. As student satisfaction has often been linked to student persistence, such surveys give administrators valuable insights into how the institution's quality is perceived by different stakeholders, assisting in institutional strategic planning and goal setting.^[23]^ A study conducted in a dental college in Saudi Arabia in 2020 concluded that the teaching staff and students’ satisfaction levels with the curriculum were significantly associated with their perception that the curriculum produces competent graduates.^[24]^ Many studies have reported on the students’ opinion, assessment, perception, and satisfaction of dental education in general and in subjects such as oral surgery, periodontics, and prosthodontics.^[25,26,27,28]^ No study has been reported on the satisfaction of operative dentistry teaching and curriculum in particular. This study sought to gain greater insight since operative dentistry forms a significant bulk of dental education. Student's satisfaction to teaching systems developed by academicians are generally perceived to be high and such a hypothesis needs to be verified. This study aimed to evaluate the professional training satisfaction levels of senior undergraduate students training in operative dentistry regarding the teaching and the curriculum at different levels and among genders.

## 2 Methods

This descriptive-analytic study was conducted on level 10, 11, and 12 (clinical level) students in the academic year 2019 to 20. Ethical clearance for this study was obtained from the Institutional Review Board of King Khalid University College of Dentistry. The questionnaire distributed among the students consisted of 6 domains: learning objectives, course materials, content relevance, instructor knowledge, instructor delivery and style, and facility and environment. A total of 193 students participated in the survey, of which 109 (56.48%) were male and 84 (43.52%) were female. The respondents were students in levels 10, 11, and 12 of the courses and were in the ratio of 34.2%, 32.1%, and 33.7%, respectively. Participation in the study was voluntary, and the students were briefed regarding the study. Enough time was provided to return the completed questionnaire. The participants were encouraged to ask for any clarifications. The responses were collected anonymously. The original questionnaire was developed by RAND staff, based on J Kirkpatrick, to evaluate the adult learning principles and training evaluation.^[29]^ The questionnaire used in this study was slightly modified from the original to make it compatible for operative dentistry teaching in dental education. The responses were graded as 3, 2, or 1 corresponding to Agree, Somewhat Agree, or Disagree. After the questionnaires were collected, the data were entered into SPSS 19.0 (Inc., Chicago, Ill., USA). Descriptive and analytical Chi Squared, Mann–Whitney, and Kruskal–Wallis tests were used to evaluate the professional training satisfaction level with education offered at different levels and between genders in operative dentistry teaching and curriculum. The *P* value was set at .05.

## 3 Results

The results for all 6 domains showed no statistical difference in responses in terms of gender (Table 1). Likewise, no statistical difference in responses was seen between the levels, except for Q2 in the learning domain (*P* = .008) (Table 2). It was observed that the percentage of respondents with disagreement was higher to Q2 in the learning domain as compared to the other domains among the 3 levels. The average total score for all questionnaire domains and comparison between genders showed that mean scores for each domain were closer to the maximum scores. Furthermore, there was no statistically significant difference in the average satisfaction scores between genders, (Table 3). The overall results for total satisfaction and its dimensions show that there is no statistically significant difference in average satisfaction scores among student levels. However, in the learning domain, level 10 respondents had significantly lower total satisfaction average scores than the scores of respondents in other levels (Table 4). The association between levels of satisfaction (high and low) based on demographic profile showed statistically significant differences among the 3 levels (10, 11, and 12) (*P* = .045), but no difference was seen in terms of the gender of the students (Table 5).

**Table 1:** Comparison of item responses of male and female students on the scale.

Variables	Items	Male	%	Female	%	Total	%	χ^2^	*P* value
Learning objectives	**Q1** I have understood the learning objectives of the operative course.								
	Disagree	0	0.00	0	0.00	0	0.00	0.062	.803
	Somewhat agree	21	19.27	15	17.86	36	18.65		
	Agree	88	80.73	69	82.14	157	81.35		
	**Q2** I have gained knowledge and skills consistent with the learning objectives.								
	Disagree	7	6.42	4	4.76	11	5.70	0.244	.885
	Somewhat agree	18	16.51	14	16.67	32	16.58		
	Agree	84	77.06	66	78.57	150	77.72		
	**Q3** This course has clarified my role as a student.								
	Disagree	0	0.00	0	0.00	0	0.00	0.039	.843
	Somewhat agree	7	6.42	6	7.14	13	6.74		
	Agree	102	93.58	78	92.86	180	93.26		
Course materials	**Q1** The course materials (slides, lectures, assignments, quiz, etc.) are easy to follow.								
	Disagree	5	4.59	3	3.57	8	4.15	0.124	.940
	Somewhat agree	14	12.84	11	13.10	25	12.95		
	Agree	90	82.57	70	83.33	160	82.90		
	**Q2** The complexity and level of detail of the materials are appropriate.								
	Disagree	2	1.83	0	0.00	2	1.04	1.558	.459
	Somewhat agree	5	4.59	4	4.76	9	4.66		
	Agree	102	93.58	80	95.24	182	94.30		
	**Q3** The course materials, including resources, are essential to my success in operative dentistry								
	Disagree	6	5.50	3	3.57	9	4.66	0.575	.750
	Disagree	8	7.34	5	5.95	13	6.74		
	Somewhat agree	95	87.16	76	90.48	171	88.60		
Content relevance	**Q1** I shall be able to apply what I learned during this course in future as a dentist.								
	Disagree	0	0.00	0	0.00	0	0.00	1.557	.212
	Somewhat agree	2	1.83	0	0.00	2	1.04		
	Agree	107	98.17	84	100.0	191	98.96		
	**Q2** I have obtained the necessary knowledge and skills to become a successful dentist.								
	Disagree	2	1.83	0	0.00	2	1.04	1.558	.459
	Somewhat agree	5	4.59	4	4.76	9	4.66		
	Agree	102	93.58	80	95.24	182	94.30		
	**Q3** I know where to find answers to questions that may arise in my role as a dentist.								
	Disagree	7	6.42	3	3.57	10	5.18	0.976	.614
	Somewhat agree	8	7.34	5	5.95	13	6.74		
	Agree	94	86.24	76	90.48	170	88.08		
Instructor knowledge	**Q1** My learning was enriched by the instructor's knowledge.								
	Disagree	4	3.67	2	2.38	6	3.11	0.265	.876
	Somewhat agree	4	3.67	3	3.57	7	3.63		
	Agree	101	92.66	79	94.05	180	93.26		
	**Q2** My learning was enriched by the experience of the instructor and the examples shared in the class.								
	Disagree	5	4.59	2	2.38	7	3.63	1.228	.541
	Somewhat agree	7	6.42	8	9.52	15	7.77		
	Agree	97	88.99	74	88.10	171	88.60		
Instructor delivery and style	**Q1** I was well engaged during the operative course.								
	Disagree	5	4.59	3	3.57	8	4.15	0.325	.850
	Somewhat agree	6	5.50	6	7.14	12	6.22		
	Agree	98	89.91	75	89.29	173	89.64		
	**Q2** I found it easy to be actively involved during the learning process.								
	Disagree	5	4.59	3	3.57	8	4.15	0.123	.940
	Somewhat agree	9	8.26	7	8.33	16	8.29		
	Agree	95	87.16	74	88.10	169	87.56		
	**Q3** I had ample opportunity to ask questions and receive answers.								
	Disagree	2	1.83	2	2.38	4	2.07	0.074	.964
	Somewhat agree	5	4.59	4	4.76	9	4.66		
	Agree	102	93.58	78	92.86	180	93.26		
	**Q4** I had ample opportunity to practice and demonstrate the skills that I leant.								
	Disagree	6	5.50	9	10.71	15	7.77	1.797	.407
	Somewhat agree	7	6.42	5	5.95	12	6.22		
	Agree	96	88.07	70	83.33	166	86.01		
	**Q5** I was comfortable with the pace of the operative sessions in the course.								
	Disagree	7	6.42	9	10.71	16	8.29	1.163	.559
	Somewhat agree	9	8.26	7	8.33	16	8.29		
	Agree	93	85.32	68	80.95	161	83.42		
	**Q6** I was comfortable with the length of the operative sessions in the course.								
	Disagree	9	8.26	8	9.52	17	8.81	0.245	.885
	Somewhat agree	11	10.09	7	8.33	18	9.33		
	Agree	89	81.65	69	82.14	158	81.87		
Facility and environment	**Q1** I found the operative laboratory and the operative clinic free of distractions and conducive to study								
	Disagree	1	0.92	0	0.00	1	0.52	0.804	.669
	Somewhat agree	3	2.75	2	2.38	5	2.59		
	Agree	105	96.33	82	97.62	187	96.89		
	Total	109	100.0	84	100.0	193	100.0		

**Table 2:** Comparison of item responses on the scale based on level.

Variables	Items	10th level	%	11th level	%	12th level	%	χ^2^	*P* value
Learning objectives	**Q1** I have understood the learning objectives of operative course.								
	Disagree	0	0.00	0	0.00	0	0.00	4.015	.134
	Somewhat agree	15	22.73	14	22.58	7	10.77		
	Agree	51	77.27	48	77.42	58	89.23		
	**Q2** I have gained knowledge and skills consistent with the learning objectives.								
	Disagree	9	13.64	1	1.61	1	1.54	13.664	.008^∗^
	Somewhat agree	11	16.67	13	20.97	8	12.31		
	Agree	46	69.70	48	77.42	56	86.15		
	**Q3** The course has clarified my role as a student.								
	Disagree	0	0.00	0	0.00	0	0.00	2.967	.227
	Somewhat agree	7	10.61	4	6.45	2	3.08		
	Agree	59	89.39	58	93.55	63	96.92		
Course materials	**Q1** The course materials (slides, lectures, assignments, quiz, etc.) are easy to follow.								
	Disagree	5	7.58	2	3.23	1	1.54	6.937	.139
	Somewhat agree	12	18.18	8	12.90	5	7.69		
	Agree	49	74.24	52	83.87	59	90.77		
	**Q2** The complexity and level of detail of the materials are appropriate.								
	Disagree	0	0.00	2	3.23	0	0.00	4.942	.293
	Somewhat agree	4	6.06	3	4.84	2	3.08		
	Agree	62	93.94	57	91.94	63	96.92		
	**Q3** The course materials, including resources, are essential to my success in operative dentistry								
	Disagree	5	7.58	2	3.23	2	3.08	7.021	.135
	Disagree	8	12.12	2	3.23	3	4.62		
	Somewhat agree	53	80.30	58	93.55	60	92.31		
Content relevance	**Q1** I shall be able to apply what I learned during this course in the future as a dentist.								
	Disagree	0	0.00	0	0.00	0	0.00	1.029	.598
	Somewhat agree	1	1.52	1	1.61	0	0.00		
	Agree	65	98.48	61	98.39	65	100.0		
	**Q2** I have obtained the necessary knowledge and skills to become a successful dentist.								
	Disagree	1	1.52	1	1.61	0	0.00	1.038	.904
	Somewhat agree	3	4.55	3	4.84	3	4.62		
	Agree	62	93.94	58	93.55	62	95.38		
	**Q3** I know where to find answers to the questions that may arise in my role as a dentist.								
	Disagree	4	6.06	4	6.45	2	3.08	3.383	.496
	Somewhat agree	7	10.61	3	4.84	3	4.62		
	Agree	55	83.33	55	88.71	60	92.31		
Instructor knowledge	**Q1** My learning was enriched by the instructor's knowledge.								
	Disagree	2	3.03	3	4.84	1	1.54	1.397	.845
	Somewhat agree	3	4.55	2	3.23	2	3.08		
	Agree	61	92.42	57	91.94	62	95.38		
	**Q2** My learning was enriched by the experience of the instructor and the examples shared in the class.								
	Disagree	4	6.06	2	3.23	1	1.54	2.268	.687
	Somewhat agree	5	7.58	4	6.45	6	9.23		
	Agree	57	86.36	56	90.32	58	89.23		
Instructor delivery and style	**Q1** I was well engaged during the operative course.								
	Disagree	5	7.58	1	1.61	2	3.08	5.383	.250
	Somewhat agree	6	9.09	4	6.45	2	3.08		
	Agree	55	83.33	57	91.94	61	93.85		
	**Q2** I found it easy to be actively involved in the learning process of the operative course.								
	Disagree	5	7.58	1	1.61	2	3.08	5.861	.210
	Somewhat agree	8	12.12	5	8.06	3	4.62		
	Agree	53	80.30	56	90.32	60	92.31		
	**Q3** I had ample opportunity to ask questions and receive answers during my course.								
	Disagree	3	4.55	1	1.61	0	0.00	4.070	.397
	Somewhat agree	4	6.06	2	3.23	3	4.62		
	Agree	59	89.39	59	95.16	62	95.38		
	**Q4** I had ample opportunity to practice and demonstrate skills that I had learnt.								
	Disagree	7	10.61	3	4.84	5	7.69	3.109	.540
	Somewhat agree	6	9.09	3	4.84	3	4.62		
	Agree	53	80.30	56	90.32	57	87.69		
	**Q5** I was comfortable with the pace of the operative sessions in the course.								
	Disagree	9	13.64	3	4.84	4	6.15	6.785	.148
	Somewhat agree	8	12.12	5	8.06	3	4.62		
	Agree	49	74.24	54	87.10	58	89.23		
	**Q6** I was comfortable with the length of the operative sessions in the course.								
	Disagree	10	15.15	3	4.84	4	6.15	6.515	.164
	Somewhat agree	8	12.12	5	8.06	5	7.69		
	Agree	48	72.73	54	87.10	56	86.15		
Facility and environment	**Q1** I found the operative laboratory and the operative clinics free of distractions and conducive to study.								
	Disagree	1	1.52	0	0.00	0	0.00	3.495	.479
	Somewhat agree	3	4.55	1	1.61	1	1.54		
	Agree	62	93.94	61	98.39	64	98.46		
	Total	66	100.0	62	100.0	65	100.0		

∗ *P* < .05.

**Table 3:** Comparison of total satisfaction and its dimensions based on gender by Mann–Whitney *U* test.

Components	Summary	Male	Female	Total	*Z* value	*P* value
Learning objectives (Total score possible = 9)	Mean	8.45	8.49	8.47	−0.571	.567
	SD	1.03	1.08	1.05		
Course materials (Total score possible = 9)	Mean	8.51	8.62	8.56	−0.259	.794
	SD	1.16	1.02	1.10		
Content relevance (Total score possible = 9)	Mean	8.70	8.82	8.75	−0.393	.693
	SD	0.92	0.58	0.79		
Instructor knowledge (Total score possible = 6)	Mean	5.73	5.77	5.75	−0.028	.977
	SD	0.83	0.73	0.79		
Instructor delivery and style (Total score possible = 18)	Mean	16.94	16.76	16.87	−0.165	.868
	SD	2.52	2.65	2.57		
Facility and environment (Total score possible = 3)	Mean	2.95	2.98	2.96	−0.156	.876
	SD	0.25	0.15	0.21		
Total satisfaction (Total score possible = 54)	Mean	51.29	51.44	51.36	−0.302	.762
	SD	5.93	5.14	5.59		

**Table 4:** Comparison of total satisfaction and its dimensions by level using Kruskal–Wallis test.

Components	Summary	10th level	11th level	12th level	*H* value	*P* value
Learning objectives	Mean	8.23	8.47	8.71	4.369	.037^∗^
(Total score possible = 9)	SD	1.31	0.94	0.79		
Course materials	Mean	8.33	8.60	8.75	0.826	.363
(Total score possible = 9)	SD	1.29	1.08	0.85		
Content relevance	Mean	8.68	8.73	8.85	0.190	.663
(Total score possible = 9)	SD	0.86	0.91	0.57		
Instructor knowledge	Mean	5.70	5.74	5.82	0.026	.873
(Total score possible = 6)	SD	0.89	0.83	0.63		
Instructor delivery and style	Mean	16.21	17.23	17.18	0.067	.796
(Total score possible = 18)	SD	3.30	1.97	2.10		
Facility and environment	Mean	2.92	2.98	2.98	0.001	.973
(Total score possible = 3)	SD	0.32	0.13	0.12		
Total satisfaction	Mean	50.08	51.74	52.29	3.314	.069
(Total score possible = 54)	SD	7.11	4.62	4.39		

∗ *P* < .05.

**Table 5:** Association between levels of satisfaction based on demographic profile.

Profile	Levels of satisfaction					χ^2^	*P* value
	Low level^∗∗^	%	High level^∗∗∗^	%	Total		
Level							
Level 10	21	31.82	45	68.18	66	6.199	.045^∗^
Level 11	13	20.97	49	79.03	62		
Level 12	9	13.85	56	86.15	65		
Gender							
Male	23	21.10	86	78.90	109	0.201	.653
Female	20	23.81	64	76.19	84		
Total	43	22.28	150	77.72	193		

∗ *P* < .05.

∗∗ Scores less than or equal to mean is considered as low level.

∗∗∗ Scores above the mean considered as high level.

## 4 Discussion

Students at 3 different levels of the operative dentistry course in Saudi Arabia were participants of this survey on their professional training satisfaction regarding the course's teaching and curriculum. These students had already undergone preclinical training and were in clinical training. Clinical-based education is a multi-factorial process wherein students implement the theoretical knowledge they gain in preclinical training on patients. Dentistry is an essential field of medical science, and hence, enhancing the quality of clinical dental education improves people's oral/dental health.

In the Kingdom of Saudi Arabia, most colleges follow the semester-type curriculum. Each year has 2 semesters, each comprising 14 weeks of actual teaching and 4 weeks of practical/clinical and final theory exams. Operative dentistry starts at level 4 of the dental course. Levels 4 to 6 are primarily preclinical courses where the students are introduced to the materials they will be using and work in simulated laboratories learning different cavity design preparations and restorations with different restorative materials. The Level 4 course is primarily involved in introducing the students to the instruments and materials. The Level 5 course is solely dedicated to amalgam cavity preparations and restorations, whereas Level 6 involves composite cavity preparations and restorations. In addition, they have E-Learning assignments on the recent advances in material sciences and techniques. Levels 10, 11, and 12 involve students implementing their skills on patients under their supervisors’ direct supervision. The curriculum in Level 10 requires the students to work with amalgam restorations on patients compulsorily. Level 11 and 12 students work with composite cavity preparations and restorations and are also trained in esthetic restorations.

Modern education for women in the Arab world is considered recent in its history. In countries like Saudi Arabia, modern education for women is a 20th-century event.^[30,31]^ The universities in Saudi Arabia admit male and female students, but they have different campuses.

During their professional training, male students treat male patients, and female students treat female patients at their respective campuses. Parahoo et al investigated whether gender was a factor in measuring overall student satisfaction in universities in the Gulf region.^[32]^ It was found that the 2 genders displayed a difference in the factors influencing their satisfaction. A study in 2017 investigated the potential barriers to the professional development of female dentists in Saudi Arabia and suggested recommendations to minimize the effects of these barriers.^[33]^ Hence, in the present study, the level of professional training satisfaction between male and female students was also investigated.

Each course has its learning objectives specified and described at the beginning of the course. During the first lecture, the learning objectives were explained to the students, and the same was evaluated at the end of the semester by testing whether students obtained the requisite knowledge and skills consistent with the learning objectives and were clear about what is expected of them. In the current study, it was observed that the respondents agreed that most of the learning objectives were met during the course, except for questions regarding knowledge and skills. Level 10 students whose skills were primarily based on Amalgam cavity preparations and restorations had a statistical difference, which was significant (*P* = .008) (Table 2). The role of amalgam in operative dentistry today is being debated. The level of awareness of the present generation of students makes them question the need to learn a skill that they might not practice in their professional careers.

During the course, the students are exposed to various teaching materials and strategies such as lectures, power point presentations, and assessment criteria such as quizzes, online assignments, and continuous evaluation of their preclinical work/clinical work. These need to be evaluated and assessed to understand whether they are consistent with the learning objectives to achieve the intended goal. The results of this study indicated that all 3 items under the Course Material domain showed that neither the level nor the gender led to any statistical difference in responses (Tables 1 and 2).

After training, it is critical to understand the implications of how the student applies his training as a dentist in society. The learning process aims to produce an independent thinking dentist who will be able to apply the necessary knowledge and skills using his or her rationale as a successful dentist. The relevance of the course is reflected when the intended goals are achieved. The results of this study indicated that most respondents agreed that course training increased their level of confidence to work as an independent dentist. All 3 items under the Course Relevance domain showed no statistical difference in responses regardless of gender or level. A majority of the respondents (>88%) agreed that the course was relevant, as seen from the training (Tables 1 and 2).

Instructors play an essential role in shaping the attitude and enthusiasm of the students. The instructor's knowledge and skills, along with his or her experience, is a vital component of the teaching process, especially in clinical sciences. Students often get influenced and motivated by the instructor. The students were exposed to a fixed group of instructors on a rotational basis during the semester. In the Kingdom of Saudi Arabia, as of now, most universities have teachers from various parts of the world who have been trained in their own countries. The teachers undergo continuous orientation programs of the university to update, understand, and comply with the teaching and assessment patterns. A study evaluated the satisfaction rate of clinical dentistry students with clinical teaching in Kermanshah University of Medical Sciences between 2015 and 2016.^[34]^ They stated that maximum cooperation of the professors and accurate implementation of the educational curriculum could have a significant effect on increasing student satisfaction. It is essential to understand the instructors’ impact on professional training satisfaction at different levels and between genders. This study showed that most of the respondents (>80%) had a favorable opinion about the role of instructors. There was no statistical difference in terms of gender or level (Tables 1 and 2).

Since operative dentistry is a clinical subject, the importance of clinical facilities and the environment must be emphasized. The clinical setup, availability of the latest materials, and instruments and their utilization during the clinical training period play a vital role in students’ education. Most respondents in this study agreed (>90%) that the operative dentistry clinics’ Facility and conduciveness were satisfactory. There was no statistical difference based on gender or level (Tables 1 and 2). However, it is interesting to note that satisfaction was more significant at a higher level of students.

Analyzing the association between the levels of satisfaction from the perspective of gender and level, it was interesting to observe that there was a statistical difference in terms of the level of students (Table 5). The 2 levels of satisfaction (low and high) were based on mean scores. If the scores are less than or equal to the mean, it is considered a low level, and the scores are above the mean, it is considered a high level. Level 10 students recorded a lower level of satisfaction (31.82%), whereas levels 11 and 12 recorded a high level of satisfaction (68.18%). Level 10 students are the junior-most level of students among the respondents. The amount of exposure to the professional field is less as compared to the higher level of students. The clinical work level 10 students perform under supervision on the patients is more basic than levels 11 and 12. Level 10 students must perform a certain number of amalgam cavity preparations and restorations during their clinical training. The students’ attitudes toward amalgam restorations and the difficulty in convincing the patients to undergo treatment with amalgam have been documented.^[35,36]^ This factor may have resulted in a lower level of satisfaction among the level 10 students (Table 5). There was no statistical difference in the gender group (Table 5). However, it is interesting to observe that the level of satisfaction increased in students’ higher levels, indicating a greater level of professional training satisfaction.

Dentistry is a clinical major in which adequate skills and training are highly crucial in graduate students’ performance, promoting oral and dental health systems. There are some limitations of this study. The educational system, equipment available, performance of tutors, availability of clinical material, and students’ expectations might be different in dental schools and hence the results of this study may not be generalizable to other dental schools. This study did not assess the reasons for shortcomings in professional training, as it was not in the purview of this study. This could be another limitation of the present study. The professional satisfaction of senior graduates who have passed and are into practice could also add more depth to our understanding of the effectiveness of the operative teaching curriculum.

## 5 Conclusions

This study focused primarily on operative dentistry teaching and the relevance of the present curriculum offered in the Kingdom of Saudi Arabia. Feedback and student satisfaction would be an essential tool in assessing and improving education. The study findings show that most students of Levels 10, 11, and 12 were satisfied with the teaching and operative dentistry curriculum. However, regarding the skills obtained during the course, the question arises whether the need to teach the principles of amalgam cavity preparation and restoration, which the students might not utilize at all in their professional careers, is still valid. Re-evaluation of the curriculum, focusing on present and future needs, or limiting the amalgam exercises to preclinical training, may be suggested. This will give the students more time to practice clinical setup skills, which will help them professionally. The level of satisfaction was also observed to be higher at higher levels of the course. However, this study provides an insight for policymakers in the future to improve upon. To achieve higher levels of satisfaction, it is recommended to set up course development committees with effective student representation and participation to fill up the lacunae that might be present regarding professional training. With rapid innovation in terms of materials and techniques in operative dentistry, the curriculum needs to be updated and continuously assessed by students to understand their satisfaction as they are the final recipients of the educational process and the future of healthcare in any nation.

## Acknowledgments

The author would like to thank Dr. S B Javali for the statistical analysis and Dr. Shashit Shetty Bavabeedu to help acquire the data. The author would also like to thank all the dental students who participated in the study.

## Author contributions

**Conceptualization:** Mohammed A. Alqarni.

**Data curation:** Mohammed A. Alqarni.

**Formal analysis:** Mohammed A. Alqarni.

**Investigation:** Mohammed A. Alqarni.

**Methodology:** Mohammed A. Alqarni.

**Project administration:** Mohammed A. Alqarni.

**Writing – original draft:** Mohammed A. Alqarni.

**Writing – review & editing:** Mohammed A. Alqarni.
